# A first‐in‐human, randomized, double‐blind, single‐ and multiple‐dose, phase I study of recombinant human thymosin β4 in healthy Chinese volunteers

**DOI:** 10.1111/jcmm.16693

**Published:** 2021-08-04

**Authors:** Xinghe Wang, Long Liu, Lu Qi, Chunpu Lei, Pu Li, Yu Wang, Chen Liu, Haihong Bai, Chengquan Han, Yinjian Sun, Jincan Liu

**Affiliations:** ^1^ Phase I Clinical Trial Centre Beijing Shijitan Hospital Capital Medical University Beijing China; ^2^ Beijing Northland Biotech. Co., Ltd. Beijing China

**Keywords:** adverse event, anti‐drug antibody, intravenous, myocardial infarction, pharmacokinetics, recombinant human thymosin β4, safety, terminal clearance, tolerability

## Abstract

The study evaluated the safety, tolerability, pharmacokinetics (PK) and anti‐drug antibody (ADA) of the recombinant human thymosin β4 (NL005) for single and multiple intravenous injections in healthy subjects. Seven cohorts, with 54 healthy subjects, were given a single intravenous dose of NL005 or placebo and were observed for 28 days. The cohorts received ascending doses of either 0.05, 0.25, 0.5, 2.0, 5.0, 12.5 or 25.0 μg/kg in the single‐dose trial. A total of 30 healthy subjects were randomly enrolled in the multiple‐dose trial, and 3 cohorts (0.5, 2.0 and 5.0 μg/kg) were administered once human thymosin β4 daily for 10 days and observed for 28 days. The adverse events were mild to moderate in intensity. There were no dose‐limiting toxicities or serious adverse events. The plasma concentration, maximum peak concentration (*C*_max_) and AUC of each dose group increased with the increase in the dose. The tendency of terminal clearance in each dose group was consistent, and there was no obvious accumulation after continuous administration. Thus, the drug can be concluded to be well tolerated and safe in healthy people and suitable for use in a clinical study for the treatment of acute myocardial infarction.

## 1 INTRODUCTION

Thymosin beta 4 (thymosin β4, Tβ4) is a peptide comprising 43 amino acids that abounds in the human body. It has anti‐inflammatory property promoting angiogenesis in the ischaemic sites and anti‐apoptotic and anti‐fibrotic property promoting tissue repair.^1^ In the recent years, studies have explored the clinical indications of Tβ4, especially in treating ischaemic brain or cardiovascular diseases and have made a breakthrough.^2^, ^3^

In cardiovascular research, Tβ4 plays an important role in improving blood supply to ischaemic myocardium, reducing myocardial injury, promoting myocardial tissue repair and improving microvascular obstruction,^4^, ^5^ providing a new therapeutic target for myocardial infarction. Presently, similar products have completed the phase I clinical study of interventions for acute myocardial infarction in the United States. The study results show that Tβ4 is well tolerated and safe with no dose‐limiting toxicity and serious adverse events. Also, no tumorigenesis was observed within 6 months.^6^, ^7^, ^8^

NL005 (recombinant human thymosin β4,rh‐Tβ4) comprised 44 amino acids and was generated via restructuring of products by the Beijing northland biotech co., Ltd, using genetic engineering. It has biological activities identical to that of the natural Tβ4. It has undergone complete preclinical pharmacy, pharmacodynamics, pharmacokinetics and safety evaluation, and the preclinical studies have shown NL005 to be stable, of high‐quality, safe and effective for increasing the heart function, improving the heart configuration and increasing collateral circulation. It imparts anti‐apoptotic property and cell oxidation resistance with no abnormalities during the synthesis. Further developments are however required for harnessing Tβ4 in treating acute myocardial infarction clinically.

## 2 MATERIALS AND METHODS

### 2.1 Trial design

This study was a single‐centre, double‐blind, randomized, placebo‐controlled, dose‐increasing trial. It was divided into single intravenous administration (SAD) and multiple intravenous administration (MAD) (clinicaltrials.gov: CTR20170766; CTR20181784). The SAD was conducted from 11 July 2017 (first patient in) to 20 June 2018 (last patient out). MAD was conducted from 16 October 2018 (first patient in) to 1 April 2019 (last patient out). SAD screened 279 subjects and randomly enrolled 54, MAD screened 242 subjects, 30 were randomly enrolled. This study was approved by the National Medical Products Administration (approval number: 2015L05215), followed by the Helsinki principle and Good clinical practice, approved by the National Medical Ethics Committee of Beijing, Shijitan Hospital, Capital Medical University. The main purpose of this study was to evaluate the safety and tolerability, pharmacokinetics (PK) and anti‐drug antibody (ADA).

In the SAD, 54 healthy subjects were enrolled and seven cohorts (0.05, 0.25, 0.5, 2, 5, 12.5 and 25 μg/kg) were administered. Considering the safety, 0.05 and 0.25 μg/kg cohorts (2 subjects per cohort) were used as the pre‐test groups, half consisting of males and half of females, and were administered NL005 once. The other five cohorts of subjects (10 subjects per cohort) were administered one dose of NL005 or placebo in a 4:1 ratio.

In the MAD, 30 healthy subjects were enrolled and three cohorts (0.5, 2, 5 μg/kg) were formed, half consisting of males and half of females. Each cohort (10 subjects per cohort) was given NL005 or placebo at a ratio of 4:1 for 10 consecutive days.

### 2.2 Subject eligibility

Male (weight ≥50 kg) and female (weight ≥45 kg) volunteers (19 ≤ BMI ≤ 28 kg/m^2^) between 18 and 50 years and in good health, with no underlying medical conditions that would place them at risk, were evaluated for the study participation. All the subjects provided informed consent. The exclusion criteria included clinically significant abnormalities, smoking history (the previous 3 months > 5 cigarettes per day, or did not stop smoking during the experiment), drinking history (the previous 3 months > 2 units per day (1 unit = 360 mL beer, 150 mL wine or 45 mL liquor with 40% alcohol content) or alcohol test positive) and history of substance abuse or drug screening test positive, pregnancy and lactation plan, ADA positive and allergic to protein preparations and biological products.

### 2.3 Procedure

The subjects for SAD were admitted to the clinical pharmacology unit on day 1 of the study and were discharged from the unit on day 5. The NL005 or placebo was administered as a single dose on day 1. The safety parameters including vital signs, physical examination, CBC, chemistries, myocardial injury markers, coagulation function and ECGs were recorded on the days 2, 5, 14 and 28. The tumour markers were examined on day 14. From the 2nd to 7th cohorts, the pharmacokinetic samples were obtained on the day 1, collected from each subject before dose administration; immediately and 15, 30 and 45 minutes post‐dose, and at 1, 1.5, 2, 2.5, 3, 4, 6, 8 and 12 hours post‐dose. Blood samples were collected for determining the serum antibody for NL005 at screening day 14 and day 28. Evaluation of the treatment‐emergent adverse events was performed throughout the study.

The subjects were admitted to the pharmacology unit on day 1 and remained confined to the unit through day 11 of the MAD. The NL005 or placebo was administered as a single intravenous dose starting from day 1 and then daily for 10 days. The safety parameters including vital signs, physical examination, CBC, chemistries, myocardial injury markers, coagulation function and ECGs were performed intermittently on the days 5, 11 and 28 of the evaluation of adverse events. The tumour markers were examined on day 14 and day 28. The pharmacokinetic samples were obtained from day 1 to 10 from each subject before dosing immediately, 30 minutes, 1, 1.5, 2, 2.5, 4, 6, 8 and 12 hours post‐dose. The blood samples were collected for determining the serum antibody against NL005 at screening, days 14 and 28. Subjects were discharged on day 11 and returned for the outpatient evaluations on days 14 and 28.

Investigational products (vials of Tβ4 0.1 mg) or placebo (saline solution) were produced and tested by the Beijing Northland Biotechnology and stored at 2‐8℃. The placebo was indistinguishable from NL005 in shape, colour, package and label.

According to NCI CTCAE4.03, DLT in this study was defined as meeting all the following conditions within 14 days after administration: subjects developed liver, kidney, heart, psychoneurosis toxicity (≥2 grade) or other systemic adverse events (≥3 grade). The relationship was assessed to be possibly, probably or related to the study drug. Definition of maximum tolerated dose (MTD) in this study: occurrence of three or more DLTs in SAD, occurrence of four or more DLTs in MAD should terminate the test, and the previous dose of this dose is regarded as the MTD. During SAD, escalation to the next dosing cohort occurred after a blinded review of all the cohort safety data through day 14. During MAD, a safety review of all the cohort data occurred after day 28.

### 2.4 Statistical analysis

All statistical analyses were done with the SAS 9.4 (SAS Institute) in the Statistical Analysis Department, Jiaxing Taimei Medical Technology. The pharmacokinetic parameters and ADA indexes were tested and analysed by Joinn Laboratories (China) Co., Ltd. The pharmacokinetic parameters were estimated using the Phoenix WinNonlin software (Certara).

All the pharmacokinetic samples in the single and multiple dosing tests were collected, centrifuged and packed by the research centre and transported to the testing unit in the frozen state on dry ice (≤−70℃). They were tested and analysed for the plasma concentration using the validated LC‐MS/MS (Waters, UPLC‐Xevo TQ‐S) analysis method, and the quantitative lower limit is 0.22 ng/mL for the SAD and 0.3 ng/mL for MAD. The non‐compartment model (NCA) was used to calculate the pharmacokinetic parameters, and the main parameters obtained were *C*_max_, *T*_max,_ AUC, *t*_1/2,_ CL and *V*_z_.

All the ADA samples are transported to the testing unit in the cold chain under dry ice (≤−70℃) and were screened and confirmed with the indirect ELISA analysis method, and the positive samples were tested for the antibody titre.

## 3 RESULTS

### 3.1 Demographic and baseline characteristics

Fifty‐four subjects were enrolled in SAD (10 placebos, 44 NL005), half of which were males and the other half were females. Thirty subjects were enrolled in MAD (6 placebos, 24 NL005). The study volunteers had fairly consistent characteristics across all cohorts in terms of age, weight and BMI etc (Table 1).

**TABLE 1:** Demographics

	Gender		Age (y)	Race		Weight (kg)	Height (cm)	BMI (kg/m^2^)
	Male	Female		Han	Other			
Placebo (N = 10)	5	5	27.4	9	1	67.35	164.1	24.91
0.05 μg/kg (N = 2)	1	1	31.0	2	0	67.90	166.0	24.60
0.25 μg/kg (N = 2)	1	1	28.0	2	0	69.80	168.5	24.60
0.5 μg/kg (N = 8)	4	4	25.8	8	0	64.48	165.8	23.45
2.0 μg/kg (N = 8)	4	4	28.6	7	1	61.56	165.3	22.51
5.0 μg/kg (N = 8)	4	4	27.8	7	1	62.25	160.9	24.10
12.5 μg/kg (N = 8)	4	4	32.8	8	0	63.28	164.5	23.31
25.0 μg/kg (N = 8)	4	4	28.8	7	1	63.35	163.5	23.69
Placebo (N = 6)	3	3	30.7	6	0	71.68	170.7	24.58
0.5 μg/kg (N = 8)	4	4	28.1	8	0	64.58	170.6	22.14
2.0 μg/kg (N = 8)	4	4	29.1	7	1	63.06	166.3	22.74
5.0 μg/kg (N = 8)	4	4	29.9	8	0	61.61	165.4	22.54

### 3.2 Safety and tolerability

The adverse events (AEs) were coded by the MedDRA 21.0, and the relationship was assessed by CTCAE4.03, and the clinical safety was evaluated. There were no serious adverse events or dose‐limiting toxicities during the SAD. Seven of 10 (70%) placebo subjects demonstrated AEs, 25 of 44 (56.8%) Tβ4 cohorts too. All the AEs were mild and moderate, only one placebo subject experienced remission after medication for upper respiratory tract infection, and the rest experienced spontaneous remission. The incidence and severity of adverse events were equal in the Tβ4 group and the placebo group. (Table 2).

**TABLE 2:** Summary of the single‐dose adverse events

Adverse events	Placebo (N = 10)	0.05 μg/kg (N = 2)	0.25 μg/kg (N = 2)	0.5 μg/kg (N = 8)	2.0 μg/kg (N = 8)	5.0 μg/kg (N = 8)	12.5 μg/kg (N = 8)	25 μg/kg (N = 8)	Total study drug (N = 44)
Number of subjects	7	2	2	6	5	3	4	3	25
Total adverse events	12	4	3	9	10	6	8	5	45
Abnormal blood biochemical	4	1	2	1	2	1	2	0	9
Abnormal blood routine	1	3	0	1	3	1	1	0	9
Abnormal SCC	0	0	0	1	1	0	3	3	8
Abnormal routine urine	4	0	0	1	3	1	1	1	7
Abnormal other tumour markers	1	0	0	3	0	0	0	1	4
Abnormal ECG	1	0	0	2	1	0	0	0	3
Cervicitis	0	0	0	0	0	1	0	0	1
Pelvic inflammation	0	0	0	0	0	1	0	0	1
Upper respiratory infection	1	0	0	0	0	0	1	0	1
Vaginal infection	0	0	0	0	0	1	0	0	1
Phlebitis	0	0	1	0	0	0	0	0	1
Abnormal cardiac injury marker	0	0	0	0	0	0	0	0	0
Abnormal coagulation	0	0	0	0	0	0	0	0	0

There were no serious adverse events or dose‐limiting toxicities during the MAD. Five of six (83.3%) placebo subjects experienced AEs, and the experimental group incidence was 16 of 24 (66.7%). All the AEs were mild and moderate, untreated and alleviated by themselves. At 5 μg/kg, both placebo and Tβ4 groups had one case of subject shedding due to an abnormal electrocardiogram. The incidence and severity of adverse events were equal in the Tβ4 group and the placebo group (Table 3).

**TABLE 3:** Summary of multiple‐dose adverse events

Adverse events	Placebo (N = 6)	0.5 μg/kg (N = 8)	2.0 μg/kg (N = 8)	5.0 μg/kg (N = 8)	Total study drug (N = 24)
Number of subjects reporting	5	5	6	5	16
Total adverse events	11	10	16	13	39
Abnormal routine urine	2	1	6	1	8
Abnormal ECG	5	0	1	7	8
Abnormal blood routine	0	2	2	2	6
Abnormal blood biochemical	1	2	2	0	4
Abnormal tumour markers(SCC)	2	2	0	2	4
Hypertriglyceridaemia	1	1	2	0	3
Abnormal cardiac injury marker	0	0	1	0	1
Abnormal coagulation	0	2	0	0	2
Transient amaurosis	0	0	0	1	1
Blood vessel puncture site bruising	0	0	1	0	1
Sinus bradycardia	0	0	1	0	1

### 3.3 Pharmacokinetics

**FIGURE 1:**
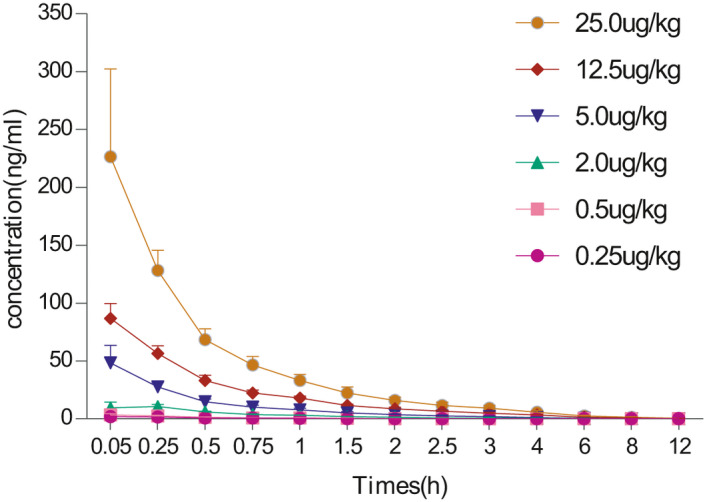
The mean ± SD of the plasma concentrations (ng/mL) of Tβ4 following single intravenous administration to healthy human subjects

**FIGURE 2:**
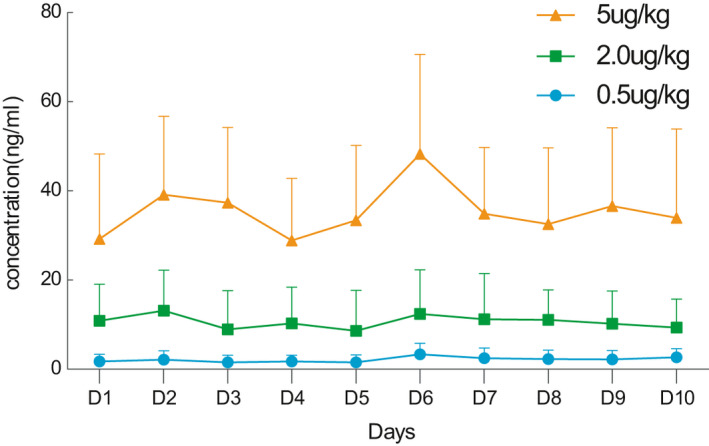
The mean ± SD of the plasma concentrations (ng/mL) of Tβ4 following multiple intravenous administration to healthy human subjects

In the SAD, the mean plasma concentration increases proportionally with the increase in the dose and drug administration time ranging from 0.25 to 25 μg/kg (Figure 1). The *C*_max_, AUC_0‐t_ and AUC_0‐∞_ demonstrated a trend of linear dynamic characteristics, but the conclusion of linear pharmacokinetic characteristics remains elusive (Table 4). The mean plasma concentration, *C*_max_ and AUC, each increased proportionally with dose, over the range of doses studied in the MAD. After continuous administration for 10 days, before each administration, the plasma drug concentration dropped to the lower limit of the dose, the tendency of terminal clearance was relatively consistent, and no obvious accumulation was observed for artesunate between the dosage of 0.5‐5.0 μg/kg after continuous administration (Figure 2).

**TABLE 4:** Summary of the main pharmacokinetic parameters for single/multiple administration

Time	Dose	*C*_max_ (ng/mL)	AUC_0_ (h*ng/mL)	AUC_0_ (h*ng/mL)	*T*_max_ (h)	*t*_1/2_ (h)	CLss (mL/h/kg)	*V*_z_ (mL/kg)
Single	0.25 μg/kg	1.99	1.26	1.47	0.05	0.5	175.985	119.925
	0.5 μg/kg	3.581	2.419	2.838	0.05	1.019	188.161	256.771
	2.0 μg/kg	11.709	11.129	12.075	0.25	1.833	169.556	432.453
	5.0 μg/kg	48.406	32.278	33.054	0.05	1.383	155.368	305.674
	12.5 μg/kg	86.896	73.604	74.863	0.05	1.92	167.883	462.041
	25.0 μg/kg	230.065	155.153	156.461	0.05	2.084	163.555	490.481
Multiple	0.5 μg/kg	3.351	1.75	2.135	216.05	0.568	257.066	192.256
	2.0 μg/kg	11.698	10.031	11.008	216.05	1.281	198.609	346.686
	5.0 μg/kg	38.243	25.607	26.513	216.05	1.413	205.993	410.639

### 3.4 The anti‐drug antibody of NL005

A total of 108 ADA samples (D14/D28) were collected from 54 subjects in SAD, and except for one positive sample, all the others were negative, and the positive detection rate was 1/108. Two exfoliated subjects were removed, and out of 28 subjects (56 ADA samples) in MAD, except for two positive samples, all the others were negative, and the positive detection rate was 1/28. All the positive samples became negative after follow‐up.

## 4 DISCUSSION

The pharmacokinetic results of both SAD and MAD showed a proportional increase in the mean plasma concentration, *C*_max_ and AUC each, with the increasing dose. Among the cohorts, the *T*_max_ was from 3 to 15 minutes, and *t*_1/2_ was from 0.5 to 2.08 hours; and it was consistent with the reports on chemical Tβ4 products.^4^ In the MAD, continuous administration for 10 days demonstrated a relatively consistent trend of terminal clearance with no obvious accumulation after continuous administration. The anti‐drug antibody results indicate that NL005 does not produce resistant antibodies. For samples with positive anti‐drug antibody in the follow‐up period, the values of the test indices were just above the positive criterion at the first test; however, when the titres were measured, the detection indices were below the positive criterion, which suggests that systematic error caused the difference. The fact that all the positive samples turned negative after follow‐up suggests that this product did not produce resistant antibodies.

There were no serious adverse events or dose‐limiting toxicities observed during the SAD and MAD. All the AEs were mild and moderate, untreated and alleviated by themselves. The incidence and severity of the adverse events were equal in the Tβ4 and the placebo groups.

In this study, there was a mild transient increase in SCC after administration in both NL005 and placebo groups, such that all of them returned to normal after follow‐up. SCC is a tumour marker of epithelial hyperplasia with low sensitivity and specificity, ^9^, ^10^ and the time curve of mildly increased SCC was consistent with the concentration of this product in the blood, which was considered to be related to the mechanism of Tβ4 promoting the regeneration of epithelial cells.^11^, ^12^, ^13^ Recent research showed that Tβ4 participates in the metastasis of tumours and has a potential for tumour promotion by inducing angiogenesis and epithelial cell migration. However, some studies have shown that Tβ4 may have an anti‐tumour effect, and no tumour‐related adverse events were found in this study.^14^, ^15^, ^16^

As far as we know, this study is the first reported investigation of Tβ4, produced using recombinant gene technology in healthy subjects. This study showed that NL005 is well tolerated and safe and should be allowed to develop further drugs to treat acute myocardial infarction.

## CONFLICT OF INTEREST

The authors have no conflicts of interest to declare. Chengquan Han, Yinjian Sun and Jincan Liu are employees of the sponsor.

## AUTHOR CONTRIBUTION

**Xinghe wang:** Conceptualization (lead); Data curation (lead); Formal analysis (lead); Investigation (lead); Methodology (lead); Project administration (supporting); Resources (lead); Supervision (lead); Validation (lead); Visualization (lead); Writing‐original draft (lead); Writing‐review & editing (lead). **Long Liu:** Conceptualization (lead); Data curation (lead); Formal analysis (lead); Investigation (lead); Methodology (lead); Project administration (supporting); Resources (equal); Supervision (equal); Validation (equal); Visualization (equal); Writing‐original draft (lead); Writing‐review & editing (lead). **Lu Qi:** Conceptualization (lead); Data curation (lead); Formal analysis (lead); Investigation (lead); Methodology (lead); Project administration (supporting); Resources (equal); Supervision (equal); Validation (equal); Visualization (equal); Writing‐original draft (lead); Writing‐review & editing (lead). **Chunpu Lei:** Conceptualization (equal); Data curation (equal); Formal analysis (equal); Investigation (equal); Methodology (equal); Project administration (supporting); Resources (equal); Supervision (equal); Validation (equal); Visualization (equal). **Pu Li:** Conceptualization (equal); Data curation (equal); Formal analysis (equal); Investigation (equal); Methodology (equal); Project administration (supporting); Resources (equal); Supervision (equal); Validation (equal); Visualization (equal). **Yu Wang:** Conceptualization (equal); Data curation (equal); Formal analysis (equal); Investigation (equal); Methodology (equal); Project administration (supporting); Resources (equal); Supervision (equal); Validation (equal); Visualization (equal). **Chen Liu:** Conceptualization (equal); Data curation (equal); Formal analysis (equal); Funding acquisition (equal); Investigation (equal); Methodology (equal); Project administration (supporting); Resources (equal); Supervision (equal); Validation (equal); Visualization (equal). **Haihong Bai:** Conceptualization (equal); Data curation (equal); Formal analysis (equal); Investigation (equal); Methodology (equal); Project administration (supporting); Resources (equal); Supervision (equal); Validation (equal); Visualization (equal). **Chengquan Han:** Conceptualization (supporting); Data curation (supporting); Formal analysis (supporting); Funding acquisition (lead); Investigation (supporting); Methodology (supporting); Project administration (lead); Resources (supporting); Software (supporting); Supervision (supporting); Validation (supporting); Visualization (supporting). **Yinjian Sun:** Conceptualization (supporting); Data curation (supporting); Formal analysis (supporting); Funding acquisition (lead); Investigation (supporting); Methodology (supporting); Project administration (lead); Resources (supporting); Software (supporting); Supervision (supporting); Validation (supporting); Visualization (supporting). **Jincan Liu:** Conceptualization (supporting); Data curation (supporting); Formal analysis (supporting); Funding acquisition (lead); Investigation (supporting); Methodology (supporting); Project administration (lead); Resources (supporting); Software (supporting); Supervision (supporting); Validation (supporting); Visualization (supporting).

## Data Availability

The data that support the findings of this study are available from the corresponding author upon reasonable request.

## References

[1] Dube KN, Bollini S, Smart N, et al. Thymosin β4 protein therapy for cardiac repair. Curr Pharm Des. 2012;18:799‐806. 22236126 10.2174/138161212799277699

[2] Smart N, Risebro CA, Melville AAD, et al. Thymosin β4 induces adult epicardial progenitor mobilization and neovascularization. Nature. 2007;445:177‐182. 17108969 10.1038/nature05383

[3] Bock‐Marquette I, Saxena A, White MD, et al. Thymosin β4 activates integrin‐linked kinase and promotes cardiac cell migration, survival and cardiac repair. Nature. 2004;432:466‐472. 15565145 10.1038/nature03000

[4] Smart N, Bollini S, Dubé KN, et al. De novo cardiomyocytes from within the activated adult heart after injury. Nature. 2011;474:640‐644. 21654746 10.1038/nature10188 PMC3696525

[5] Smart N, Dube KN, Riley PR. Epicardial progenitor cells in cardiac regeneration and neovascularization. Vascul Pharmacol. 2013;58:164‐173. 22902355 10.1016/j.vph.2012.08.001

[6] Ruff D, Crockford D, Girardi G, et al. A randomized, placebo‐controlled, single and multiple‐dose study of intravenous thymosin β4 in healthy volunteers. Ann NY Acad Sci. 2010;1194:223‐229. 20536472 10.1111/j.1749-6632.2010.05474.x

[7] http://www.regenerx.com/RGN‐352

[8] https://www.clinicaltrials.gov/, Identifier: NCT01311518.

[9] Kulpa J, Wójcik E, Reinfuss M, et al. Carcinoembryonic antigen, squamous cell carcinoma antigen, CYFRA 21–1, and neuron‐specific enolase in squamous cell lung cancer patients. Clin Chem. 2002;48:1931‐1937. 12406978

[10] De Cos JS, Masa F, de la Cruz JL , et al. Squamous cell carcinoma antigen (SCC Ag) in the diagnosis and prognosis of lung cancer. Chest. 1994;105:773‐776. 8131539 10.1378/chest.105.3.773

[11] Sosne G, Hafeez S, Greenberry AL, et al. Thymosin ß4 promotes human conjunctival epithelial cell migration. Curr Eye Res. 2002;24:268‐273. 12324865 10.1076/ceyr.24.4.268.8414

[12] Philp D, Kleinman HK. Animal studies with thymosin beta4, a multifunctional tissue repair and regeneration peptide. Ann NY Acad Sci. 2010;1194:81. 20536453 10.1111/j.1749-6632.2010.05479.x

[13] Sosne G, Xu L, Prach L, et al. Thymosin beta 4 stimulates laminin‐5 production independent of TGF‐beta. Exp Cell Res. 2004;293(1):175‐183. 14729067 10.1016/j.yexcr.2003.09.022

[14] Caers J, Hose D, Kuipers I, et al. Thymosin β4 has tumour‐suppressive effects and its decreased expression results in poor prognosis and decreased survival in multiple myeloma. Haematologica. 2010;95:163‐167. 19833631 10.3324/haematol.2009.006411 PMC2805724

[15] Morita T, Hayashi K. Tumor progression is mediated by thymosin‐β4 through a TGFβ/MRTF signaling axis. Mol Cancer Res. 2018;16:880‐893. 29330296 10.1158/1541-7786.MCR-17-0715

[16] Cha H‐J, Jeong M‐J, Kleinman HK. Role of thymosin 4 in tumor metastasis and angiogenesis. J Natl Cancer Inst. 2003;95(19). 10.1093/jnci/djg100 14625258

